# Antioxidant, Antimicrobial and Phytochemical Variations in Thirteen *Moringa oleifera* Lam. Cultivars

**DOI:** 10.3390/molecules190710480

**Published:** 2014-07-18

**Authors:** Ashwell R. Ndhlala, Rofhiwa Mulaudzi, Bhekumthetho Ncube, Hafiz A. Abdelgadir, Christian P. du Plooy, Johannes Van Staden

**Affiliations:** 1 Agricultural Research Council, Vegetable and Ornamental Plants Institute (VOPI), Private Bag X293, Pretoria 0001, South Africa; E-Mails: NdhlalaA@arc.agric.za (A.R.N.); MulaudziR@arc.agric.za (R.M.); AbdelgadirH@arc.agric.za (H.A.A.); IduPlooy@arc.agric.za (C.P.P.); 2 Research Centre for Plant Growth and Development, University of KwaZulu-Natal Pietermaritzburg, Private Bag X01, Scottsville 3209, South Africa; E-Mail: NcubeB@ukzn.ac.za

**Keywords:** antimicrobial, antioxidant, free radicals, *Moringa oleifera* Lam.

## Abstract

A study was undertaken to assess variation in antioxidant, antimicrobial and phytochemical properties of thirteen *Moringa oleifera* cultivars obtained from different locations across the globe. Standard antioxidant methods including the DPPH scavenging, ferric reducing power (FRAP) and *β*-carotene-linoleic acid model were used to evaluate the activity. Variation in the antioxidant activity was observed, with TOT4951 from Thailand being the most active, with activity five times higher than that of ascorbic acid (reference compound). A different trend was observed for the activity in the FRAP and *β*-carotene-linoleic acid assays. Antimicrobial activity was tested against Gram-positive (*Staphylococcus aureus*) and Gram-negative (*Klebsiella pneumoniae*) strains using the microdilution method. Acetone extracts of all cultivars exhibited good antibacterial activity against *K. pneumoniae* (MIC values of 0.78 mg/mL). The remaining extracts exhibited weak activity against the two microorganisms. For the antifungal activity, all the extracts exhibited low activity. Variations were observed in the total phenolic and flavonoid contents. Cultivars TOT5169 (Thailand) and SH (South Africa) exhibited highest amounts of total phenolic compounds while TOT5028 (Thailand) exhibited the lowest amounts of five times lower than the highest. The information offer an understanding on variations between cultivars from different geographical locations and is important in the search for antioxidant supplementation and anti-ageing products.

## 1. Introduction

Free radical damage from reactive oxygen species (ROS) has been linked to the progressive decrease in normal function and accumulation of macromolecular damage that gradually leads to ageing [1]. The ageing process is usually accompanied by several human pathologies including diabetes, cardiovascular disorders, cancer and neurodegenerative diseases, which can also be aggravated by exposure to physiological stressors such as ROS [2]. However, variation in the ageing and onset of these diseases/disorders suggests a high degree of variability in tolerance to ROS and biological ageing in humans. 

Under manageable concentrations, ROS exert beneficiary effects to the body. However, when expressed in high levels, ROS leads to oxidative stress [1]. Protection against ROS damage depends on the expression of the antioxidant systems within the body or external supplementation of antioxidants [3]. During infections, inflammation and several other pathologies, the body defends itself from further injury by use of ROS such as the superoxide anion, hydroxyl radicals, nitric oxide and hydrogen peroxide from normal cell redox processes [4,5]. Regulation of ROS defense can be reinforced by supplementation with plant derived natural extracts and compounds, such as resveratrol; and antioxidant exogenous sources enriched with flavonoids and vitamin C from the diet and epidermal antioxidant activity from enriched cosmetics [6].

Several medicinal plants have been reported to act as sources of exogenous antioxidants. Amongst them, *Moringa oleifera*, is one of the most widely distributed species of a monogeneric family Moringaceae [7]. The tree is characterized as a fast-growing, drought-tolerant type, native to north-western India, and is widely cultivated in tropical and subtropical areas where its young seed pods and leaves are regarded as a nutritional powerhouse [8]. Several compounds have been isolated from the leaves of *Moringa oleifera* including niazirin, niazirinin, 4-[4'-O-acetyl-α-L-rhamnosyloxy) benzyl]isothiocyanate, niaziminin A and B [9,10].

Moringa has long been recognized in traditional medicine worldwide as having value both as a preventative and treatment agent of several health conditions, including the treatment of inflammation, infectious diseases, cardiovascular, gastrointestinal, haematological and hepatorenal disorders [11]. Several scientific articles has been published describing the antioxidant properties of Moringa, which can translate to its use as an anti-ageing herb [12]. However, much of the evidence remains anecdotal as there has been little actual scientific research done to support these claims.

This study was aimed at investigating the variations in the antioxidant activity using the DPPH (2,2-diphenyl-1-picrylhydrazyl) radical scavenging assay, ferric-reducing power and the ability to delay or halt the bleaching of *β*-carotene-linoleic acid in a model system as well as the antibacterial properties between thirteen *Moringa oleifera* cultivars introduced from four different geographical locations of the world. The variations in the phytoconstituents of the cultivars were also investigated using colourimetric methods.

## 2. Results and Discussion

A comparative study to determine the variation in the antioxidant, antimicrobial and phytochemical properties of extracts of thirteen *Moringa oleifera* cultivars introduced from The World Vegetable Centre (AVRDC) (Thailand), Taiwan, South Africa and United States of America was carried out. There were significant differences in the antioxidant, antimicrobial activities and phytochemical properties between the cultivars. A correlation analysis between the phytochemical content (flavonoids and total phenolics) and antioxidant activities (DPPH and β-carotene-linoleic acid) revealed insignificant but mostly weak negative correlations (*p* > 0.05), with coefficient (r) values of between −0.464 to 0.036. However, although insignificant, the negative correlations, in the context of antioxidant activities, indicate some appreciable degree of correlation between high phenolic levels and good antioxidant activities.

### 2.1. DPPH Radical Scavenging Activity

The EC_50_ values for the DPPH radical scavenging potentials of the thirteen cultivars are shown in Table 1. The widely used parameter to measure antioxidant activity is the concentration of a test sample needed to decrease the initial DPPH concentration by 50% and is denoted as EC_50_ [13]. In this study, EC_50_ values less than or equal to 70.12 µg/mL [that of ascorbic acid (reference/positive control)] were considered good activity. The radical scavenging activity of the cultivars against DPPH radicals according to the respective EC_50_ values (Table 1) were in the following descending order: TOT4951 > TOT5028 > TOT7266 > TOT4880 > TOT4100 > TOT4893 > TOT4977 > Limpopo > TOT5330 > TOT5077 > SH > CHM. The first three most active cultivars were all from the AVRDC, Thailand and the last two were the Silver Hill, South Africa cultivars. 

All the cultivar extracts showed a DPPH radical scavenging ability higher than that of a reference compound ascorbic acid (vitamin C). The most active DPPH radical scavenger, TOT4951 exhibited a scavenging ability five times more than that of ascorbic acid. The least active DPPH radical scavenger CHM, had twice the scavenging ability compared to ascorbic acid. This suggests that all the cultivar extracts tested in this study serves as better antioxidants than ascorbic acid. The experimental data reveal that all the cultivar extracts are likely to have the effect of scavenging free radicals and thus can be incorporated into cosmetics for healthy skin and/or antiageing products.

### 2.2. β-Carotene-Linoleic Acid Model System (CLAMS) Activity

The results of the delay in *β*-carotene bleaching, recorded as antioxidant activity (ANT %) and Oxidation Rate Ratio (ORR), calculated on the basis of the rate of *β*-carotene bleaching at time = 60 min are shown in Table 1. The order of antioxidant activity with respect to the protection of *β*-carotene against bleaching by the extracts with ORR values ≤ 0.05 was as follows; TOT4977 = TOT5028 = TOT4893 > SH. Lower ORR values, just like EC_50_ values, denote better antioxidant potentials. Most of the cultivar extracts performance was lower compared to that of ascorbic acid in the prevention of *β*-carotene bleaching. Several plant secondary metabolites including phenolics and flavonoids are known to possess the ability to protect certain compounds like *β*-carotene against oxidation [14].

**Table 1:** Antioxidant activity of extracts from thirteen *Moringa oleifera* cultivars introduced from different geographical world locations as determined by the DPPH scavenging assay and *β*-carotene-linoleic acid model system. *n* = 3.

Cultivar	Cultivar Origin	Antioxidant Activity		
		DPPH Scavenging ActivityEC_50_ (µg/mL)	ANT (%)	ORR
TOT4880	USA	**16.70 ± 0.00 ^a^**	37.46 ± 2.35 ^a^	0.73 ± 0.02 ^e^
TOT4977	AVRDC	**20.91 ± 0.00 ^ab^**	87.71 ± 1.81 ^efg^	**0.02 ± 0.00 ^a^**
TOT5077	AVRDC	**22.45 ± 1.70 ^ab^**	44.40 ± 1.66 ^ab^	0.51 ± 0.03 ^cde^
TOT5028	AVRDC	**16.40 ± 3.04 ^a^**	98.00 ± 0.16 ^g^	**0.02 ± 0.00 ^a^**
TOT5169	AVRDC	**26.88 ± 2.30 ^bc^**	83.50 ± 0.33 ^de^	0.16 ± 0.13 ^ab^
SH	Silver Hill (SA)	**23.96 ± 1.41 ^abc^**	94.83 ± 1.77 ^fg^	**0.05 ± 0.02 ^a^**
TOT4893	AVRDC	**20.06 ± 4.29 ^ab^**	87.71 ± 0.50 ^efg^	**0.02 ± 0.01 ^a^**
CHM	Silver Hill (SA)	**32.56 ± 6.57 ^c^**	94.04 ± 0.10 ^efg^	0.06 ± 0.00 ^ab^
TOT5330	AVRDC	**22.00 ± 2.58 ^ab^**	96.49 ± 1.16 ^g^	0.72 ± 0.45 ^e^
TOT7266	AVRDC	**16.54 ± 2.72 ^a^**	63.42 ± 1.83 ^bc^	0.27 ± 0.08 ^bc^
TOT4100	Taiwan	**16.78 ± 1.50 ^a^**	55.83 ± 1.01 ^ab^	0.39 ± 0.06 ^cd^
TOT4951	AVRDC	**14.57 ± 0.65 ^a^**	86.02 ± 0.12 ^ef^	0.24 ± 0.13 ^ab^
Limpopo	Limpopo (SA)	**21.44 ± 1.08 ^ab^**	73.59 ± 0.69 ^cd^	0.46 ± 0.21 ^de^
Ascorbic acid		71.11 ± 0.01 ^d^	81.45 ± 1.72^de^	0.19 ± 0.02 ^ab^

Cultivar extracts with EC_50_ values (<71.11 µg/mL) and written in bold are considered potent DPPH radical scavengers. The lower the EC_50_, the more rapidly the colour of DPPH radical was bleached and hence the more potent the antioxidant. ANT (%)—Antioxidant activity calculated on the basis of the rate of *β*-carotene bleaching at *t* = 60 min. ORR - Oxidation Rate Ratio at *t* = 60. The lower the ORR value, the more protective the compound/extract against *β*-carotene bleaching. Cultivar extracts with ORR values ≤0.05 and written in bold are considered potent antioxidants. SA—South Africa, AVRDC—World Vegetable Centre, Thailand. Mean values (±SE) in column with different letters are significantly different (*p* < 0.05; *n* = 3).

### 2.3. Ferric-Reducing Power Assay Activity

**Figure 1:**
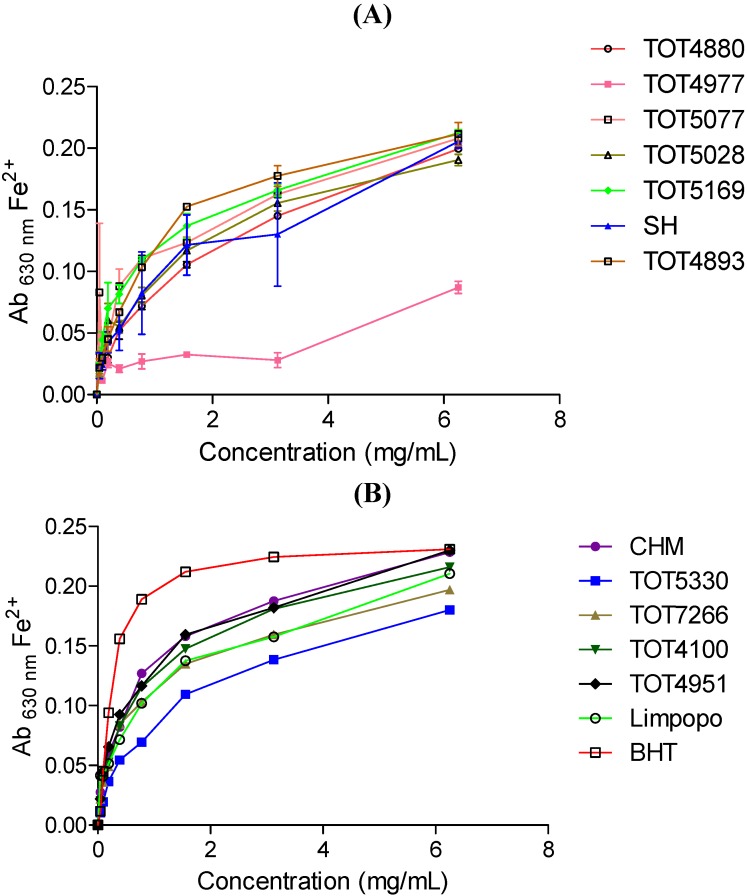
Ferric reducing activity of thirteen *Moringa oleifera* cultivars introduced from different geographical locations around the world. BHT- butylated hydroxytoluene. Increase in absorbance of the reaction mixture indicates the increase in reducing power. (**A**) TOT4880; TOT4977; TOT5077; TOT5028; TOT5169; SH; TOT4893; (**B**) CHM; TOT5330; TOT7266; TOT4100; TOT4951; Limpopo; BHT (positive control).

The abilities of the different cultivars at varying concentrations to reduce Fe^3+^ complexes in solution are presented in Figure 1 (the Figure was split into A and D to allow visibility of individual lines representing different cultivars). Strong antioxidants (reductants) reduce the Fe^3+^ complex to various shades of green and the blue ferrous form, and is characterised by higher absorbance values at λ 630 nm after the assay [14]. Reducing activity increased with the increase in the concentration of all the cultivar extracts (as expected). The reducing activity of bioactive extracts is directly associated with antioxidant activity as the reduction of the Fe^3+^ complex by the bioactive compounds is brought about by the donation of electrons. This can be translated to the reaction with ROS, thereby converting them to more stable products, terminating radical chain reactions [15]. There were significant differences in the reduction power with cultivar TOT4977 from the AVRDC, Thailand performing as the least reducing agent. All the Moringa cultivar extracts exhibited lower activities compared to butylated hydroxytoluene (BHT) used as a reference compound (Figure 1B). 

### 2.4. Antimicrobial Activity

The antibacterial and antifungal minimum inhibitory concentration (MIC) values for the thirteen Moringa cultivar extracts are presented in Table 2. The cultivar extracts with MIC values <1 mg/mL were considered as having high antibacterial activity [16] and are highlighted in bold. The extracts showed a broad spectrum of activities against *Klebsiella* *pneumoniae versus* *Candida albicans*. Of particular interest was the activity of all the acetone extracts of all cultivars against *K*. *pneumoniae* (MIC values of 0.78 mg/mL each). Similar activity was observed for ethanol extracts of TOT4880, TOT5077 and CHM cultivars against *K*. *pneumoniae*. On the other hand, TOT5077 and TOT4951 ethanol extracts exhibited similar activity against *Staphylococcus aureus*. The rest of the extracts exhibited moderate to weak activity against the two microorganisms. The acetone extracts of Limpopo, TOT4977 and TOT4893 cultivars performed better against *S. aureus* than the other acetone extracts of the other cultivars although the MIC values were below the 1 mg/mL mark. All the extracts exhibited low to moderate activity against *C. albicans*. As observed for the *S. aureus* results, the acetone extracts of Limpopo cultivar performed better than the rest of the acetone extracts of the other cultivars although the MIC value was below the 1 mg/mL target level.

**Table 2:** Antibacterial and antifungal activities (MIC) of thirteen *Moringa oleifera* cultivars introduced from different world geographical locations as determined by the microdilution method.

	Solvent								
	Acetone			EtOH			Water		
	Microorganism								
Variety	*K.p.*	*S.a.*	*C.a.*	*K.p.*	*S.a.*	*C.a.*	*K.p.*	*S.a.*	*C.a.*
TOT4880	**0.78**	3.125	3.125	**0.78**	1.56	3.125	1.56	6.25	6.25
TOT4977	**0.78**	1.56	3.125	1.56	1.56	3.125	1.56	6.25	6.25
TOT5077	**0.78**	3.125	3.125	**0.78**	**0.78**	3.125	1.56	6.25	6.25
TOT5028	**0.78**	3.125	3.125	1.56	1.56	3.125	1.56	6.25	>6.25
TOT5169	**0.78**	3.125	3.125	1.56	1.56	3.125	1.56	6.25	6.25
SH	**0.78**	3.125	3.125	1.56	1.56	3.125	1.56	6.25	6.25
TOT4893	**0.78**	1.56	3.125	1.56	1.56	6.25	1.56	>6.25	3.125
CHM	**0.78**	3.125	3.125	**0.78**	1.56	6.25	1.56	6.25	6.25
TOT5330	**0.78**	3.125	3.125	1.56	1.56	6.25	1.56	6.25	3.125
TOT7266	**0.78**	3.125	3.125	1.56	1.56	6.25	1.56	6.25	3.125
TOT4100	**0.78**	3.125	3.125	1.56	1.56	6.25	1.56	6.25	3.125
TOT4951	**0.78**	3.125	3.125	1.56	**0.78**	3.125	6.25	6.25	3.125
Limpopo	**0.78**	1.56	1.56	1.56	1.56	3.125	6.25	3.125	3.125

*K*. *p*. = *Klebsiella pneumoniae*; *S*. *a*. = *Staphylococcus aureus*; *C.a. = Candida albicans*; EtOH—70% ethanol extracts; Values in bold are considered to be very active (MIC < 1 mg/mL); MIC values for neomycin was 0.8 × 10^−3^ mg/mL for *K.p.* and 1.6 × 10^−3^ mg/mL for *S.a.* and for Amphotericin B was 9.77 × 10^−3^ mg/mL for *C.a.*

*Klebsiella pneumoniae* is a Gram-negative baterium, found in the normal flora of the mouth, skin, and intestines. Apart from pneumonia, *K. pneumoniae* can also cause infections in the skin, urinary tract, lower biliary tract and open-cut/surgical wounds. The bacterium has been reported to be resistant to multiple antibiotics. This is because it belongs to the extended-spectrum beta-lactamase (ESBL)-producing strains. ESBL-producing strains have persistently shown multi-resistance to many broad-acting antibiotics such as aminoglycosides, fluoroquinolones, tetracyclines, chloramphenicol, and trimethoprim/sulfamethoxazole [17]. The fight against ESBL-producing strains such as *K. pneumoniae* is emerging as an important challenge in both synthetic and natural product development [18]. The cosmetic industry cannot be left out. This is because production of topical cosmetics for skin care products with extracts that are active against ESBL-producing strains such as *K. pneumoniae* could offer a stepping stone in the battle. The active extracts of Moringa presented in Table 2 could be incorporated into cosmetics for that purpose.

*Staphylococcus aureus*, a member of the Firmicutes, is an important Gram-positive coccus bacterium that cause diseases in humans [19]. It is frequently found in the human respiratory tract and on the skin. Although *S. aureus* is not always pathogenic, it is a common cause of skin infections such as pimples, boils, cellulitis folliculitis, carbuncles, scalded skin syndrome and abscesses [20]. Cosmetic products with antibiotic activity especially against *S. aureus* are useful as antiageing agents in that they maintain a healthy skin. They could also offer the solutions to the recent emergence of antibiotic-resistant strains called methicillin-resistant *Staphylococcus aureus* (MRSA) which are fast becoming a global problem [21].

### 2.5. Total Phenolics and Flavonoid Content

**Figure 2:**
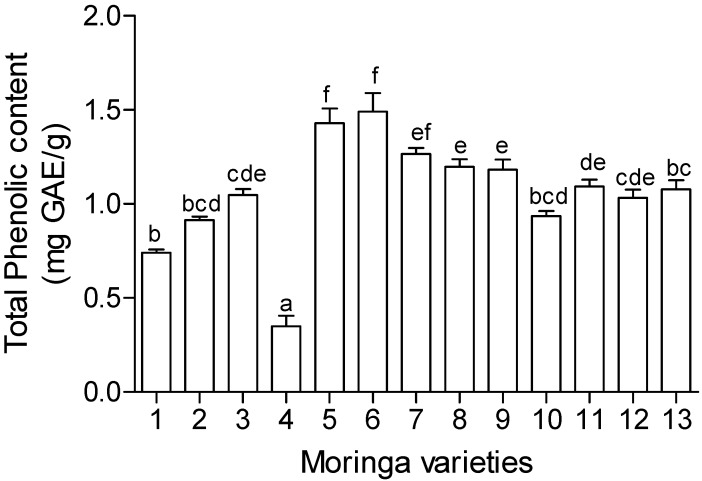
Total phenolic content of thirteen *Moringa oleifera* cultivars introduced from different geographical locations around the world. 1. TOT4880; 2. TOT4977; 3. TOT5077; 4. TOT5028; 5. TOT5169; 6. SH; 7. TOT4893; 8. CHM; 9. TOT5330; 10. TOT7266; 11. TOT4100; 12. TOT4951; 13. Limpopo. Values expressed as gallic acid equivalent (GAE) per gram of plant extracts. Mean values (±SE) on bar graphs with different letters are significantly different (*p* < 0.05; *n* = 6).

**Figure 3:**
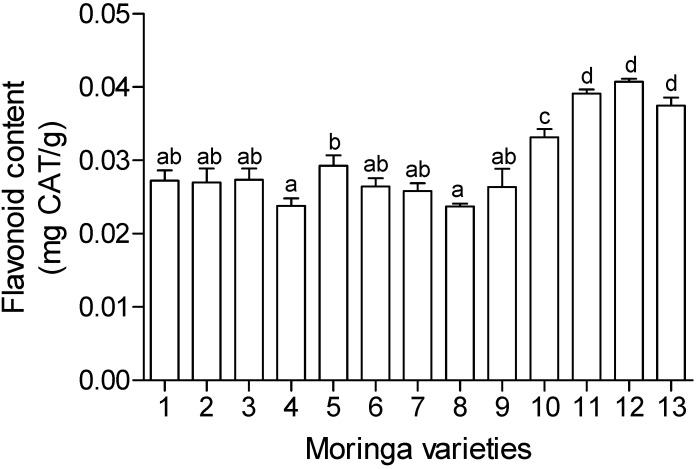
Flavonoid content of thirteen *Moringa oleifera* cultivars introduced from different geographical locations around the world. 1. TOT4880; 2. TOT4977; 3. TOT5077; 4. TOT5028; 5. TOT5169; 6. SH; 7. TOT4893; 8. CHM; 9. TOT5330; 10. TOT7266; 11. TOT4100; 12. TOT4951; 13. Limpopo.Values expressed as catechin equivalents (CAT) per gram of plant extracts. Mean values (±SE) on bar graphs with different letters are significantly different (*p* < 0.05; *n* = 6).

The phytochemical analysis carried out in this study included total phenolics and flavonoid content and the results are presented in Figure 2 and Figure 3. There were different levels of phenolic compounds detected in the different cultivars. The same trend was also noticed in the flavonoid content. Cultivars TOT5169 and SH exhibited the highest amounts of total phenolic compounds while TOT5028 exhibited the lowest amounts of at least five times lower than the highest. Different levels of expression of plant secondary metabolites like phenolic compounds suggests the differences in the ability of different cultivars in establishing themselves in new environments. Change in environment may exert stress on the plants and this may result in expression of more plant secondary metabolites. However, bioactivity cannot always be matched with the amount of phenolic compounds. For example TOT5169 and SH exhibited the highest phenolic content amongst the tested cultivars (Figure 2) but the two cultivars showed moderate antioxidant activity (Table 1) while TOT5028 exhibited the lowest amounts of phenolics but showered a better antioxidant activity compared to the former two.

Plants with high phenolic composition, including tannins, are regularly used as a basis for the production of valuable synthetic compounds such as pharmaceuticals, cosmetics, or more recently, nutraceuticals [22]. At lower concentrations, phytochemical compounds have beneficial effects such as antioxidant effects. Phenolic levels of up to 4% of dry matter have been shown to contribute positively in diets. However, as stated above, the beneficiary effects still depend on the nature and type of phenolic compounds present. In some instances, depending on the chemistry of the phenolic compounds, phytochemicals at higher concentrations (>4% in dry matter) may have negative physiological effects such as neurological problems, reproductive failure, goiter, gangrene and in lower animals may lead to death [23].

In cosmetics and antiageing products, phenolics enhance rapid skin and tissue regeneration and have demonstrated antiseptic effects (antibacterial and antifungal) [24]. In skin burns and wound healing, phenolics–protein complexes forms a film which limits fluid loss and forms a physical barrier to damaged tissue, insulating them from bacterial infection or chemical damage [25,26].

## 3. Experimental

### 3.1. General

2,2-Diphenyl-1-picrylhydrazyl (DPPH), *β*–carotene and neomycin were obtained from Sigma–Aldrich (Sigma Chemical Co., Steinheim, Germany); butylated hydroxytoulene (BHT) and potassium ferricyanide from BDH Chemicals Ltd (Poole, England, UK); trichloroacetic acid, ascorbic acid, polyoxyethylene sorbitan monolaurate (Tween 20), ferric chloride (FeCl_3_) and methanol from Merck KGaA (Darmstadt, Germany). All other chemicals used were obtained locally and were of analytical grade.

Thirteen *Moringa oleifera* Lam. cultivars collected from different geographical locations in the world were cultivated at the Agricultural Research Council (ARC) experimental farm, Roodeplaat, Pretoria. The trial layout was in a randomized block design fashion with all the cultivars receiving the same management practices of no fertilizers and watering thrice a week. Eight cultivars were introduced from the World Vegetable Centre (AVRDC) in Thailand (TOT4893, TOT4951, TOT4977, TOT 5028, TOT5077, TOT5169, TOT5330 and TOT7266), one cultivar from Taiwan (TOT4100), one cultivar from USA (TOT4880) and three cultivars from South Africa [Silver Hill (SH), CHM and Limpopo].

### 3.2. Sample Preparation

Fresh leaf samples from each of the thirteen *Moringa oleifera* cultivars were separately oven dried at 50 °C for 48 h. Dried plant materials were ground into powders and extracted non-sequentially (1:20 w/v) with 50% aqueous methanol, acetone, 70% aqueous ethanol (EtOH) and water in an ultrasonic bath for 1 h. The extracts were filtered under vacuum through Whatman’s No. 1 filter paper. The extracts in 50% aqueous methanol, acetone and EtOH were concentrated under pressure using a rotary evaporator at 30 °C and completely dried under a stream of air while water extracts were freeze-dried. Fresh extracts of 50% aqueous methanol were used in the phytochemical analysis and antioxidant assays while the acetone, EtOH and water extracts were used in the antimicrobial assays (resuspended in 70% ethanol).

### 3.3. Bioassays

#### 3.3.1. DPPH Radical Scavenging Activity

The DPPH radical scavenging assay was done as described by Karioti *et al.* [27] with modifications. Extracts of each cultivar (15 µL) at varying concentrations; 0.065, 0.26, 0.52, 1.04, 6.25, 12.5, 25 and 50 mg/mL; in triplicate, were diluted in absolute methanol (735 µL) and added to freshly prepared DPPH solution (750 µL, 50 µM in methanol) to give a final volume of 1.5 mL in the reaction mixture. The above processes were done under dimmed light and incubated at room temperature for 30 min in the dark. Absorbance of the reaction mixtures were read at 517 nm using a UV-vis spectrophotometer (Varian Cary 50, Varian Australia Pvt LTD, Sydney, Australia), with methanol as the blank solution. A standard antioxidant, ascorbic acid at varying concentrations as follows; 5, 10, 20, 40, 80 µM; were used as a positive control. A solution with the same chemicals without extracts or standard antioxidants but with absolute methanol served as the negative control. The assay was repeated twice. The free radical scavenging activity (RSA) as determined by the decolouration of the DPPH solution was calculated according to the formula:

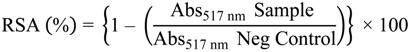

where Abs_517_ sampleis the absorbance of the reaction mixture containing the resuspended cultivar extract or positive control solution, and Abs_517_ Neg control is the absorbance of the negative control. The EC_50_ (effective concentration) values, representing the amount of extract required to decrease the absorbance of DPPH by 50% was calculated from the percentage radical scavenging activity.

#### 3.3.2. Ferric-Reducing Power Assay

The ferric reducing power of the cultivar extracts was determined based on the method by Lim *et al.* [28] with modifications. Extracts of each resuspended cultivar (50 µL) at 6.25 mg/mL and the positive control (BHT dissolved in methanol) was added to a 96 well microtiter plate in triplicate and two-fold serially diluted down the wells of the plate. To each well, 40 µL potassium phosphate buffer (0.2 M, pH 7.2) and 40 µL potassium ferricyanide (1% in phosphate buffer, w/v) were added. The microtiter plate was covered with foil and incubated at 50 °C for 20 min. After the incubation period, 40 µL trichloroacetic acid (10% in phosphate buffer, w/v), 150 µL distilled water and 50 µL FeCl_3_ (0.1% in phosphate buffer, w/v) were added. The microtiter plate was re-covered with foil and incubated at room temperature for 30 min. The ferric-reducing power assay involves the reduction of the Fe^3+^/ferricyanide complex to the ferrous (Fe^2+^) form. Absorbance of the formed Fe^2+^ was measured at 630 nm using a microtitre plate reader (Opsys MR^TM^, Dynex Technologies Inc., Palm City, FL, USA). The ferric-reducing power of the cultivar extracts and ascorbic acid were expressed graphically by plotting absorbance against concentration. The assay was repeated twice.

#### 3.3.3. *β*-Carotene-Linoleic Acid Model System (CLAMS)

The delay or inhibition of *β*-carotene and linoleic acid oxidation was measured according to the method described by Amarowicz *et al.* [29] with modifications. *β*-carotene (10 mg) was dissolved in 10 mL chloroform in a brown Schott bottle. The excess chloroform was evaporated under vacuum, leaving a thin film of *β*-carotene near to dryness. Linoleic acid (200 µL) and Tween 20 (200 µL) were immediately added to the thin film of *β*-carotene and mixed with aerated distilled water (497.8 mL), giving a final *β*-carotene concentration of 20 µg/mL. The mixture was further saturated with oxygen by vigorous agitation to form an orange coloured emulsion. The emulsion (4.8 mL) was dispensed into test tubes to which 200 µL of the resuspended cultivar extracts at 6.25 mg/mL or butylated hydroxytoulene (BHT) (6.25 mg/mL) were added, giving a final concentration of 250 µg/mL in the reaction mixtures. Absorbance for each reaction was immediately (*t* = 0) measured at 470 nm and incubated at 50 °C, with absorbance of each reaction mixture being measured every 30 min for 180 min. Tween 20 solution was used to blank the spectrophotometer. The negative control consisted of 50% methanol in place of the sample. The rate of *β*-carotene bleaching was calculated using the following formula:

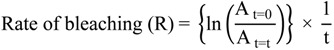

where *A_t=0_* is the absorbance of the emulsion at 0 min; and *A_t=t_* is the absorbance at time *t* (90 min; any point on the curve can be used for the calculation). The calculated average rates were used to determine the antioxidant activity (ANT) of the respective herbal preparations, and expressed as percent inhibition of the rate of *β*-carotene bleaching using the formula:

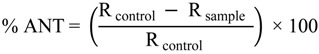

where R_control_ and R_sample_ represent the respective average *β*-carotene bleaching rates for the control and cultivar extract, respectively. Antioxidant activity was further expressed as the oxidation rate ratio (ORR) based on the equation:

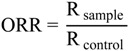



#### 3.3.4. Antibacterial Microdilution Assay

Minimum inhibitory concentration (MIC) values for antibacterial activity of the cultivar extracts were determined using the microdilution bioassay in 96-well (Greiner Bio-one GmbH, Frickenhausen, Germany) microtitre plates [30] except that 100 µL of each resuspended extract (25 mg/mL), in EtOH was two-fold serially diluted with sterile distilled water, in duplicate down the microtitre plate for each of the two bacteria used. Water, acetone and EtOH were included as negative and solvent controls. Neomycin was used as a positive control. The screening was done in triplicate and repeated twice for each extract. Two bacterial strains were used; one Gram-positive (*Staphylococcus aureus* ATCC 12600) and one Gram-negative (*Klebsiella pneumoniae* ATCC 13883).

#### 3.3.5. Antifungal Microdilution Bioassay

The antifungal activity (MIC) of the cultivar extracts against *Candida albicans* (ATCC 10231), a diploid fungus which exists in the form of a yeast, were evaluated using the microdilution assay [30] modified for an antifungal assay [31] except that 100 µL of each resuspended (in EtOH) plant extract (50 mg/mL), were two-fold serially diluted with sterile distilled water, in duplicate down the microtitre plate. Water, acetone and EtOH were included as negative and solvent controls. Amphotericin B was used as a positive control. The screening was done in triplicate and repeated twice for each extract.

#### 3.3.6. Determination of Total Phenolics and Flavonoids

The amounts of total phenolics in plant samples were determined using the Folin Ciocalteu (Folin C.) assay for total phenolics as described by Makkar [32] and modified by Ndhlala *et al.* [33]. Gallic acid was used as a standard. Flavonoids were quantified using the vanillin-HCl assay as described by Hagerman [34] with modifications [33]. Catechin was used as a standard.

### 3.4. Statistical Analysis

The data was subjected to one-way analysis of variance (ANOVA) using IBM Statistical Package for the Social Sciences (SPSS) v21.0 for Windows (Chicago, IL, USA). Significantly different means were separated using Duncan’s multiple range tests (*p* < 0.05).

## 4. Conclusions

A comparative antioxidant, antimicrobial and phytochemical analysis of thirteen *Moringa oleifera* cultivars introduced from World Vegetable Centre (AVRD) (Thailand), Taiwan, South Africa and United States of America was carried out. There were variations in the observed activity amongst the different cultivars in both the antioxidant assays and the antimicrobial assays. Variations were also observed in the phytochemical levels of the different cultivars. However, there was no direct correlation between the bioactivity and the levels of total phenolics and/or flavonoids. High DPPH scavenging activity, Fe^2+^ reducing ability and antimicrobial activity were observed in most of the cultivars. High phenolic expression in some cultivars may suggest different adaptation abilities of the cultivars to different environments. The data obtained here offer an understanding on the variations of different cultivars from different parts of the world. The information is important in the search for health care and antiageing products. It is desirable to carry out further studies to determine the effects of mixing some cultivars with other plant species used in cosmetics such as *Aloe* and determine if there is any improvement in bioactivity due to synergistic actions. It is also important to carry out safety studies to determine mutagenic and cytotoxicity properties of these cultivars as well as determining the stability and bioavailability of the natural products when used in cosmetics.
